# Bioactive Polyoxymethylene Composites: Mechanical and Antibacterial Characterization

**DOI:** 10.3390/ma16165718

**Published:** 2023-08-21

**Authors:** Paulina Kaczor, Patrycja Bazan, Stanisław Kuciel

**Affiliations:** Chair of Materials Engineering and Physics, Cracow University of Technology, 31-155 Kraków, Poland; paulina.kaczor@doktorant.pk.edu.pl (P.K.); stask@mech.pk.edu.pl (S.K.)

**Keywords:** polyoxymethylene, copper oxide, titanium oxide, zinc oxide, silver nanoparticles

## Abstract

The aim of this study is to analyze the strength and antibacterial properties of composites based on structural polyoxymethylene. The base material was modified with the most used antibacterial additives, such as silver nanoparticles, copper oxide, zinc oxide, and titanium oxide. Basic strength and low-cycle fatigue tests were conducted to determine the dissipation energy of the material. The composites were also tested for antibacterial properties against two strains of bacteria: *Escherichia coli* ATCC 8739 and *Staphylococcus aureus* ATCC 6538. Strength properties showed no significant changes in the mechanical behavior of the tested composites against the matrix material. The best antibacterial additive was the addition of titanium oxide nanoparticles, providing 100% efficacy against *Escherichia coli* and almost 100% biocidal efficacy against *Staphylococcus aureus*. The other antibacterial additives showed biocidal efficacy of about 30–40% against the unmodified material. The added value of the work is the consistency in the methodology of testing materials modified with antibacterial additives, as well as the same compactness of the introduced additives. This study makes it clear which of the introduced additives has the highest biocidal activity.

## 1. Introduction

Technical thermoplastic composites are widely used in many industries, e.g., automotive, electrotechnical, robotics, production automation, and aerospace [1]. This is closely related to the many advantages of these materials, such as low density, high stiffness and strength, good insulation properties, and corrosion resistance. In addition, these composites can be recycled, and the low weight allows for the reduction in CO_2_ emissions for the transport industry, which is very important for the environment and helps to achieve goals related to sustainable development [2]. Polyoxymethylene (POM) is an engineering thermoplastic mainly used in precision parts in the engineering and automotive industries. It is characterized by high stiffness and hardness, a low coefficient of friction, excellent dimensional stability, and low water absorption. In addition, it is relatively easy to process, and the manufactured elements are characterized by a smooth and shiny surface [3].

Because of its excellent mechanical properties due to its high crystalline phase content [4] and high chemical resistance, polyoxymethylene is widely used in many fields, such as the automotive industry, electrical and electronics industries, mechanical engineering, and the medical sector [5]. In the medical industry, it is used in aerosol system components, surgical pumps, atomizers, orthodontic device components, plug connectors, surgical instrument handles, automatic syringes, inhalers, breast pumps, food transporters, and medical devices exposed to high mechanical stress, such as automatic insulin injectors [6].

Currently, materials with antibacterial properties are being sought, especially for biomedical applications. Pathogenic bacteria are constantly evolving, increasing their resistance to previously known antibiotics [7]. Research on composites with antimicrobial properties offers the possibility of increasing the efficiency of elimination of pathogenic bacteria. The most common additives used to improve the biocidal properties of polymers are mainly metal nanoparticles (e.g., nano-Ag, nano-Au, nano-Cu, nano-Se, nano-Ag-Au, and nano-Ag-Ti) [8,9] and metal oxides (e.g., Ag_2_O, ZnO, CuO, TiO_2_, SiO_2_, and MgO) [8,10]. According to the literature data, nanosilver particles are the most used modifier to improve these properties. Research by Zeng et al. on composites with this additive showed increased antibacterial effectiveness already after the addition of about 2% Ag [11]. Mie et al. also used nano-Ag with a size of 19 nm. The studies showed high potential against Gram-negative bacteria [12]. The type, shape, and size of nanoparticles significantly affect the antimicrobial activity of composites [13,14]. Studies conducted on composites based on polypropylene with the addition of silver nanoparticles and microparticles have shown that decreasing the size of nanoparticles increases biocidal efficacy. This behavior is related to the greater surface area of nanoparticles per unit volume of the composite [15]. Similar observations were reported by Ber et al., where smaller particles showed higher activity against Gram-positive bacteria [16].

Materials modified with antibacterial additives are most often tested against the two most popular groups of bacteria, which are Gram-negative (G−) bacteria such as *Escherichia coli* (ATCC 28739) and Gram-positive (G+) bacteria such as *Staphylococcus aureus* (ATCC 6538). In addition, in the literature, tests were also carried out for the following strains of bacteria and fungi: *Candida albicans* ATCC 14053 and *Pseudomonas aeruginosa* (ATCC 10145), *Aureobasidium pullulans* var. *melanigenum* (ATCC 15233) and *Asperillus brasiliensis* (ATCC 9642), and *Sclerotium rolfsil* and *Fusarium oxysporum*) [17,18].

Antiseptic studies of polymer composites with zinc oxide and titanium oxide nanoparticles for dental applications were conducted and confirmed antimicrobial activity against bacteria in the oral cavity [19,20]. Padmavathy et al. also showed that the antimicrobial activity of ZnO is higher with additives of smaller particle size [21]. Azam et al. described the inhibitory effect of CuO nanoparticles against Gram-positive and Gram-negative bacteria. However, they noted that ZnO showed more excellent antimicrobial activity than CuO [22].

Nanoparticles not only affect the biocidal properties of composites but also can affect the composite structure and mechanical properties. Pusnik Cresnar et al. described the effect of the addition of nanoparticles of Ag (~10 nm in size), ZnO (<10 nm), and TiO_2_ (3–5 nm) on the crystallization and thermodynamic transformations of polylactide. It was shown that nanoparticles of metals and metal oxides added in small amounts (0.5–2.5 wt%) did not act as additional nuclei agents but only accelerated the crystallization process. At the same time, the crystalline fraction did not increase [23]. Ding et al. analyzed the impact of the nano-ZnO addition on PLA properties. Initially, an improvement in strength properties was noted with the addition of up to 2 wt%, while above 3 wt% and 5 wt%, the mechanical properties began to decrease along with the increase in the filler content. It was also observed that the addition of nano-ZnO affected the crystalline phase content in the composite and the rate of crystallization [24].

On the other hand, studies of composites based on polyoxymethylene by Zeng et al. showed that the degree of crystallinity (Xc) increased with an increase in the amount of nano-Ag particles (from 0 to 2% by weight) [25]. Wacharawichanant et al., in their work, analyzed the effect of the addition of nano-ZnO on the mechanical properties of the POM/nano-ZnO composite. The mechanical properties of the composites were better than those of the unmodified materials. The results showed an increase in Young’s modulus and strain at break [24]. Similar results were obtained with POM/TiO_2_ nanocomposites (Wacharawichanant et al.). The study showed an increase in mechanical properties up to 1 wt% and a drastic decrease at more than 1 wt% TiO_2_ addition [26,27].

This work aims to respond to the market demand for construction materials with appropriate mechanical and antibacterial properties. Composites based on polyoxymethylene with the addition of metal nanoparticles and metal oxides were produced. Silver (Ag), titanium oxide (TiO_2_), zinc oxide (ZnO), and copper oxide particles with different sizes were selected as antibacterial additives. Mechanical and biocidal properties were assessed. This type of research will allow the production of advanced materials to meet market demand and expand the knowledge of antibacterial additives introduced into polymeric materials. The main problem addressed in this article is the effectiveness of antibacterial additives. An amount of 2% by weight of each of the additives was incorporated into the polyoxymethylene. This value was selected based on published scientific papers suggesting a beneficial effect on strength and antibacterial properties. Exceeding this value in most cases causes a decrease in properties; furthermore, an important aspect is the problem of comparing the effectiveness of individual antibacterial additives with each other. Even though there are available studies on the antibacterial properties of zinc oxide, titanium oxide, and copper in a polyoxymethylene matrix, the methodologies used are usually different (method, measurement conditions, and environmental conditions) and biocidal activity is determined in a different way; hence, drawing correct conclusions is relatively difficult. They suggest certain properties; however, an unambiguous statement about which additive is the best is impossible, and any further assumptions and actions aimed at introducing the material may be wrong. Therefore, in this work, the most used antibacterial additives were compared using the same sample preparation methods and the same additive content, material processing, as well as research methodologies. These types of results clearly indicate the level of effectiveness of individual additives by comparing them with each other.

## 2. Materials and Methods

### 2.1. Materials Used and Sample Preparation

Polyoxymethylene (POM) is a technical material with good mechanical properties. For the manufacture of composites, a polyoxymethylene copolymer with a trade name (POM-C; Tarnoform 500 CE) was selected as the base material. POM was produced by Celanese Corporation, Irving, TX, USA, and provided by Grupa Azoty Compounding sp. z.o.o., Tarnow, Poland. Selected characteristic properties of the test materials are listed in Table 1.

Standard dumbbell and bar samples of the base material and its composites were made according to ISO 3167:2014-09 [28] using KM 40–125 Winner Kraus Maffei (Germany). The samples for strength tests were made using the injection method. The individual components of the composite were weighed and mixed in small portions to ensure the best homogenization of the components. Then, each sample was introduced into the cylinder to plasticize the material and inject it into the mold. Table 2 presents the applied processing parameters. A description of the manufactured samples is shown in Table 3. Standard mechanical samples were used for strength tests. For antibacterial tests, the samples had dimensions of 2 cm × 2 cm. The material was taken from the paddle samples from the grip part so that the tests were carried out on materials produced with the same parameters as the materials subjected to strength tests. The samples were cut using a milling machine.

### 2.2. Antibacterial Properties

Biocidal tests were carried out against two different strains of microorganisms: *Escherichia coli* ATCC 8739 and *Staphylococcus aureus* ATCC 6538. The microbiological resistance was tested using static contact in conditions of constant humidity. Each sample was subjected to UV sterilization for 30 min to eliminate potential contamination that may disturb the obtained results. Then, an inoculum of the microorganism from a 24 h culture was applied to each sample. Samples prepared in this way were placed in an incubator at 37 °C for 24 h in constant humidity conditions (approx. 70%). After the predetermined incubation time, the samples were placed in sterile flasks containing sterile phosphate buffer (0.25 M) and shaken for 5 min at 180 rpm to isolate viable microbial cells. Then plates with growth medium were inoculated using the serial dilution method and incubated again at 37 °C for 24 h. The same procedure was used for control samples without antibacterial additives. After incubation, antimicrobial efficacy was determined as the percent reduction in viability of microorganisms relative to control samples according to Formula (1):

(1) $$R=\frac{I_{w-}-I_{p}}{I_{w}}*100\%$$

where $I_{w}$—starting inoculum concentration of the microorganism (jtk/mL); and *I_p_*—concentration of the microorganisms after contact with the test sample (jtk/mL).

The higher the degree of reduction in the viability of the microorganism, the greater the biocidal effect. Each composition was tested three times.

### 2.3. Mechanical Analysis

The static tensile test was carried out according to (PN-EN ISO 527—type 1:2012) [29] on an MTS Criterion Model 43 universal testing machine (MTS System Corp., Eden Prairie, MN, USA) with an MTS axial extensometer. The measuring base was set to 100 mm, and the test speed was equal to 5 mm/min. The three-point flexural test (PN-EN ISO 178:2011) [30] was carried out using a Shimadzu AGS-X 10 kN (Kyoto, Japan) testing machine with TRAPEZIUM-X software, https://www.shimadzu.com/an/products/materials-testing/uni-ttm-software/trapezium-x/index.html. The distance between supports was set to 64 mm; test speed was 10 mm/min. The Charpy impact test was performed using a Zwick/Roell HIT5.5P hammer (Ulm, Germany) according to PN-EN ISO 179-1:2010 [31]. Measurements were examined on unnotched specimens.

Mechanical hysteresis loops were obtained by dynamic testing on a Shimadzu AGS-X 10 kN (Kyoto, Japan) testing machine with TRAPEZIUM-X software for dissipation energy analysis. The samples were subjected to cyclic loading and unloading at 10 mm/min speed. The applied force corresponded to 60% of the maximum force needed to break the sample, determined during the tensile test.

### 2.4. Water Absorption

Water absorption was conducted by the gravimetric method following the PN-EN ISO 62:2008 [32] standard. The weight was measured using an electronic Ohaus Adventurer laboratory balance (Parsippany, NJ, USA).

After weight measurements, the water absorption $N_{w}$ was calculated from Formula (2):

(2) $$N_{w}=\frac{m_{2}-m_{1}}{m_{1}}\cdot 100\%$$

where $N_{w}$—the weight water absorption (%); $m_{1}$—mass of the sample before being placed in the solution (g); and $m_{2}$—weight of the sample after being removed from the solution and dried (g).

After water absorption tests, strength tests were carried out on samples after the water aging process to determine hydrothermal degradation.

## 3. Results and Discussion

### 3.1. Antibacterial Activities of Nanocomposites Based on POM

As part of the microbiological study, five types of additives were tested. The obtained results for individual strains of microorganisms indicate that the samples modified with functional additives show different biocidal effects (Table 4). The results suggest that the highest biocidal activity against both strains was demonstrated by composites with the addition of 2 wt% titanium oxide nanoparticles (*Escherichia coli* reduction rate 100%; *Staphylococcus aureus* viability reduction rate 96%), followed by relatively high antibacterial properties of composites with 2 wt% zinc oxide (*Escherichia coli*—68%; *Staphylococcus aureus*—29%). The remaining composites expressed a low, unsatisfactory degree of reduction against both strains of bacteria.

Still, the results demonstrate outstanding potential for using metal and metal oxide nanoparticles as antibacterial additives for composites based on polyoxymethylene. As a basic principle, antibacterial additives can be divided by origin into organic and inorganic. In addition, two mechanisms of action are distinguished: biocidal (killing microorganisms) and biostatic, based on limiting growth. The inorganic additives used in these studies revealed both biocidal and biostatic effects. The biocidal effect is based on using metal ions as a killing agent. In antibacterial research, silver, copper, and zinc are the most used metal ions. The biocidal process involves introducing a metal ion into the cell membrane, which damages the cell and causes it to die. The advantage of inorganic additives is their higher thermal resistance, which allows them to be applied in a wide range of polymer matrices, including those processed at very high temperatures [33]. However, inorganic additives such as zinc pyrithione (ZnPT) and silver nanoparticles (AgNano) have also been tested by Pittol et al. The work revealed that the introduced additives did not cause changes in the strength properties, which suggests minor interactions between the composite components. Antibacterial tests showed the effectiveness of the introduced additives against *Staphylococcus aureus* (*S. aureus*) and *Escherichia coli* (*E. coli*), as well as antifungal activity against *Aspergillus niger*, *Candida albicans*, and *Cladosporium cladosporioides*. The additions of ZnPT and AgNano caused reductions in over 99% of *E. coli* and *S. aureus* populations. The content of additives was 1.5% by weight [34].

The antibacterial mechanism of metal oxide nanoparticles is not precisely known and described. A significant impact results from the release of metal ions, which are characterized by antibacterial activity, mechanical destruction of the bacterial cell wall or membrane, and oxidative stress, and the degree of bacterial reduction can be affected by many aspects, such as the percentage of nanoparticles in the composite and their shape, size, and particle synthesis [35]. TiO_2_ is described in the literature as a biologically and chemically stable substance. Numerous studies have confirmed its antibacterial potential [36,37,38,39]. The antibacterial activity of titanium oxide is related to its crystalline structure, due to which TiO_2_ has a photocatalytic effect, which affects its antibacterial activity [38,39]. The antimicrobial nature of the oxide gives it the ability to generate hydroxyl radicals under the influence of UV radiation [39,40].

Other studies have shown that additives such as titanium dioxide (TiO_2_), magnesium hydroxide (Mg(OH)_2_), and aluminum hydroxide (Al(OH)_3_) were also effective against fungal growth, and the best of the mentioned additives was titanium oxide [41]. In the case of the introduced additives, the formation of reactive oxygen species (ROS), such as HO_2_, was also indicated, causing the formation of superoxide radicals, which in turn combine with peptide bonds in the cell, causing its rupture [42].

Increasing the content of titanium oxide from 2% to 4% by weight caused a slight increase in biocidal activity. In addition, the authors state that the size of the particles is extremely significant; the additives in the form of nanoparticles have a much larger contact surface with bacteria, which promotes the oxidation of the outer cell membrane, thus increasing the biocidal effects [43,44].

Filimion et al. studied the effect of different forms as metallic silver, silver salts, and nanoparticles on the antibacterial properties of composites based on hydroxypropyl methylcellulose. In their work, they indicated that the conversion of AgNPs to Ag+ and the production of active oxygen and Ag may be a likely way to inhibit the growth of microorganisms. Silver ions cause disruption of the protein structure by forming bonds with nucleophilic amino acid residues in the protein structure. Studies have also indicated differences in the impact on Gram-negative and Gram-positive bacteria, indicating the greater susceptibility of Gram-negative bacteria, which is related to differences in their structure [11].

Zeng and others [45] conducted research on polyoxymethylene modified with silver nanoparticles. The nanoparticle content was 0.1; 0.5, 1, and 2%. The test results showed that with the increase in the content of silver nanoparticles, the antibacterial properties increased, achieving the best results at a content of 2% by weight. As a mechanism of influence, interactions with cell membranes, nucleic acids, and proteins of bacterial cells were affected.

Alli et al. also tested the effect of silver nanoparticles against *Staphylococcus aureus*, *Escherichia coli*, and two plant fungi (*Sclerotium rolfsil* and *Fusarium oxysporum*). The average particle size of silver was 12.5 nm. The silver nanoparticles showed significant antibacterial activity with a distinct zone of inhibition of 30 mm and 26 mm around the discs against *E. coli* and *S. aureus*, respectively. In addition, high antifungal efficacy was demonstrated with 100% and 76.67% growth inhibition against two plant pathogens, *S. rolfsii* and *F. oxysporum*, respectively [46].

In the case of the obtained test results, no significant effect of silver nanoparticles on the activity against *E. coli* and a slight effect on the reduction in the *Staphylococcus aureus* strain (29%) was observed. The lack of biocidal activity may be associated with the formation of agglomerates, which is highly undesirable for the controlled antibacterial effect of the colloid.

### 3.2. Mechanical Properties Investigations

The design of composite materials with the required strength properties is associated with the knowledge of the factors affecting them and the relationships between the components. The main factors influencing the properties of the composite material include, among others properties of the matrix, properties of the reinforcing phase, reinforcement content, as well as its geometry (particle size, geometry, and their orientation), quality of the connection of the matrix and the reinforcing phase, as well as the conditions of the manufacturing process and related processes of crystallization or matrix shrinkage [6].

**Figure 1:**
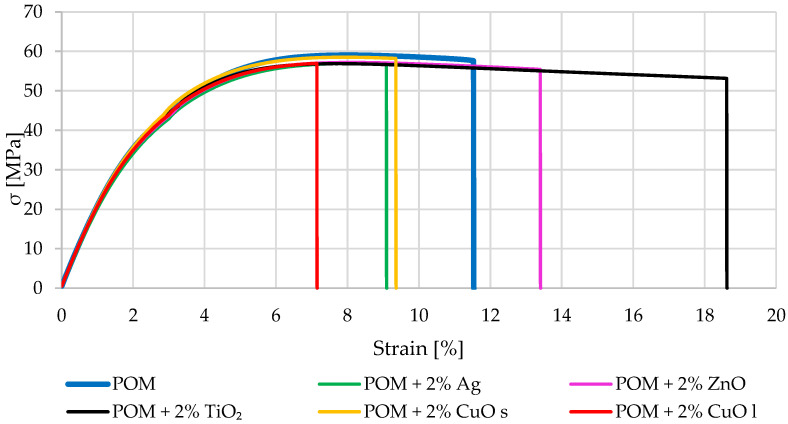
Examples of tensile curves for the tested composite material. The index s (CuO s) stands for nanoparticles, and the index l (CuO l) stands for microparticles.

The composites were subjected to static tensile, flexural, and impact tests as a mechanical test. Introducing additives in the form of particles and nanoparticles significantly impacted the strain at break value. An elongation of 12% characterized unmodified polyoxymethylene; additives such as silver and copper oxide caused a decrease in the strain at break by about 40% and reached strain at break around 9%. The introduction of titanium oxide and zinc oxide nanoparticles increased the plasticity of the tested composites. Example stretching curves are shown in Figure 1.

Introducing selected additives in the form of particles also caused a slight decrease in tensile strength and modulus of elasticity by about 2–3% compared to the unmodified material, except for materials reinforced with copper oxide. Comparisons of tensile strength, elastic modulus, and strain at break values are shown in Table 5.

**Figure 2:**
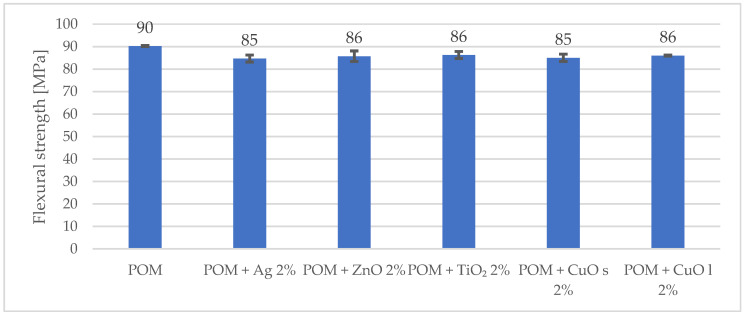
Comparison of tensile strength for composites with 2% addition of metal and metal oxide nanoparticles. The index s (CuO s) stands for nanoparticles, and the index l (CuO l) stands for microparticles.

**Figure 3:**
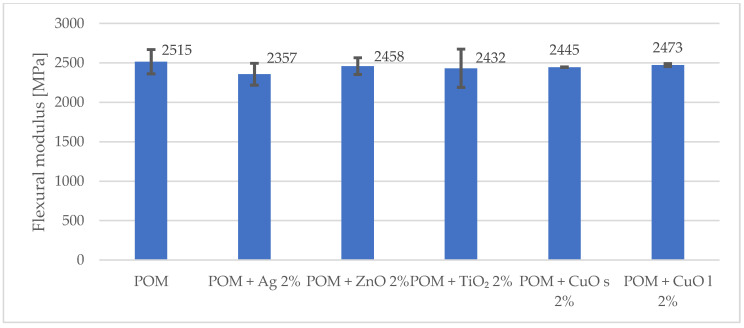
Comparison of flexural modulus for composites with 2% addition of metal and metal oxide nanoparticles. The index s (CuO s) stands for nanoparticles, and the index l (CuO l) stands for microparticles.

More significant changes in the mechanical behavior were observed during the flexural test. The flexural strength and the modulus decreased by 7% and 3%, respectively, compared to the unmodified matrix. The results are shown in Figure 2 and Figure 3.

**Figure 4:**
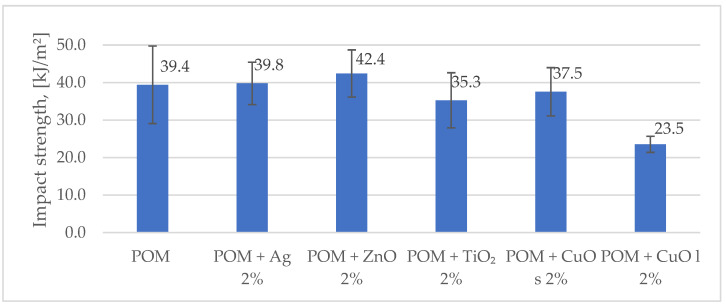
Comparison of impact strength values for the tested composite materials. The index s (CuO s) stands for nanoparticles, and the index l (CuO l) stands for microparticles.

Tests of resistance to dynamic impacts showed differentiation of results depending on the applied additive (Figure 4). Almost all the additives did not significantly affect impact strength value; only material modified with copper oxide with an average particle size of about 20 µm had reduced impact strength in relation to the rest of the compositions. This behavior may relate to the largest particle size, and the decrease in energy absorption capacity may be caused by blocking polymer chains on the incorporated particles and possible agglomeration defects.

The presented mechanical results correlate with those obtained in other studies on adding micro- and nanoparticles. He et al. and Yu et al., in their work on the impact of micro- and nanoparticles of copper introduced to polyoxymethylene, stated that the strength properties strongly depended on the added additive’s content. The range of particles in the tested composites was 10–30% by weight. The introduction of copper particles resulted in the deterioration of tribological properties. However, they noted a positive effect on the mechanical properties of composites, especially after the introduction of copper in the form of nanoparticles [47,48]. Polyoxymethylene was also modified with hydroxyapatite (HAp) nanopowder with a grain size below 100 nm (0.5–10 wt%). The results showed an increase in the flexural modulus with the increase in the content of nanoparticles. However, no effect of nanopowder on tensile strength was noted. The introduction of hydroxyapatite increased the glass transition temperature. Moreover, the activation energy was higher for the POM/HAp nanocomposites, which can be attributed to the effect of closing the polymer chains between the HAp nanoparticles, limiting their mobility. The increase in modulus of elasticity is also attributed to the limitation of the mobility of POM chains due to the presence of HAp nanoparticles, which show good adhesion to the POM matrix and their nucleating effect on POM macrochains [27,49,50,51,52]. POM composites with zinc oxide (ZnO) with a content of 1–12% by weight were also tested. Research pointed to an increase in modulus with increasing ZnO content. The tensile strength and strain at break remained unchanged at 1–4 wt% of ZnO, then decreased after increasing the ZnO content. Zinc oxide as an addition to polyoxymethylene caused a decrease in the degree of crystallinity, reducing strength properties. The maximum value of the impact strength was obtained with the content of 4 wt% zinc oxide [27].

### 3.3. Mechanical Hysteresis Loops

An interesting theory is about the determination of the initial mechanical hysteresis loops in cyclic load–unload tests. This method allows for the analysis of energy dissipation in the material. In the case of unmodified materials, energy dissipation is related to internal friction processes between macromolecules. However, in the case of composite materials, during deformation, energy is also dissipated from other effects occurring between the components of the composite (loss during pulling out/detaching the reinforcement from the matrix), in places of other defects and damages, in areas of internal stresses, etc. [52].

**Figure 5:**
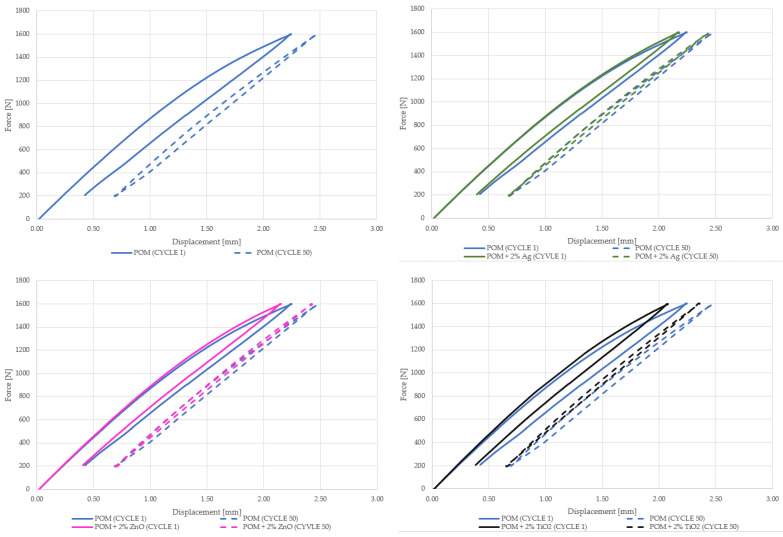

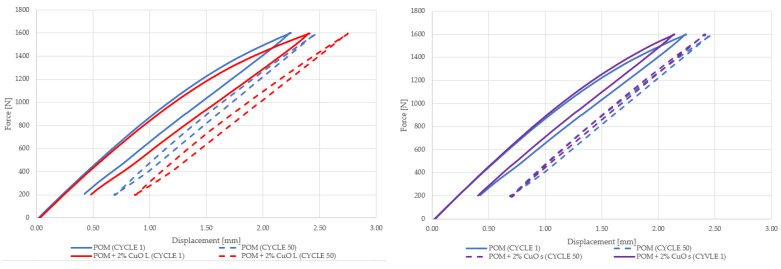
The first and fifth hysteresis loops recorded for tested materials. The index s (CuO s) stands for nanoparticles, and the index l (CuO l) stands for microparticles.

**Figure 6:**
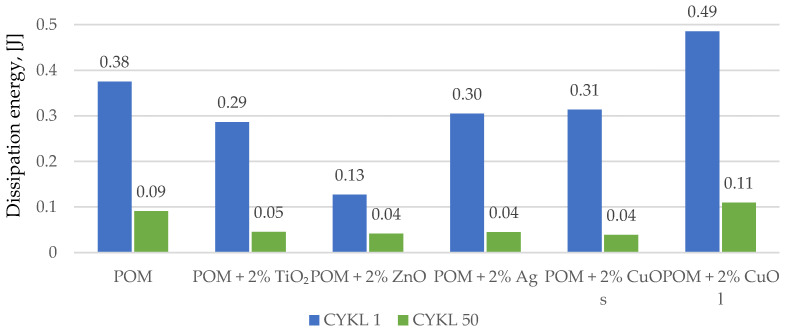
Comparison of dissipation energy values in the first and fiftieth hysteresis loops for composites based on polyoxymethylene with the addition of metal and metal oxide nanoparticles. The index s (CuO s) stands for nanoparticles, and the index l (CuO l) stands for microparticles.

**Figure 7:**
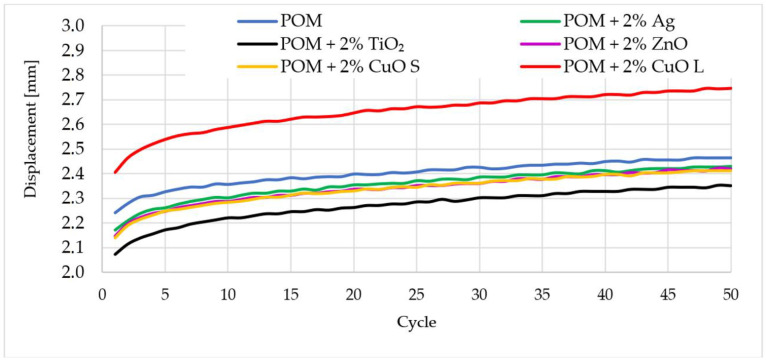
Displacement to number of cycle relation for tested materials. The index s (CuO s) stands for nanoparticles, and the index l (CuO l) stands for microparticles.

As part of this research, the first hysteresis loops were determined. Figure 5 shows the registered first and fiftieth hysteresis loops for the tested materials concerning the polymer matrix. The analysis of the obtained results showed that the introduction of silver nanoparticles did not cause significant changes in the energy dissipation capacity in relation to the polymer matrix. The recorded loops were almost identical in the first and fiftieth load cycles. Small material deformation could be observed, indicating a slight reinforcing effect. Similar conclusions can be drawn for composites with copper oxide in the form of nanoparticles and material modified with zinc oxide. Slightly different results were observed for composites with titanium oxide, where an increase in stiffness and a lower ability to dissipate energy (Figure 6) can be more clearly marked. In the case of the material modified with copper oxide particles, an increased loop area was noticed, which may suggest that the energy supplied to the system was dissipated due to the effects related to the interaction between the components–matrix–particles. In addition, dynamic creep (Figure 7) occurred in all tested materials, the highest for the material with the addition of copper oxide with larger reinforcing particles.

Cieszyński and Topoliński presented the relationship between the value of dissipated energy and the mechanical properties of composite materials. These dependencies make it possible to assess the processing process’s correctness and predict functional and long-term properties. The difference in the dissipation energy values in the first load cycles can be used to evaluate the interactions between the matrix and the reinforcement. This theory assumes that critical states of stress occur in the interfacial areas. The first load cycles may cause relaxation of the locally maximally stressed areas by their subsequent cracking, which manifests itself in the form of a hysteresis loop due to the use of the supplied energy for the cracking process [52].

The registration of several dozen hysteresis loops confirms this statement because, as mentioned before, after the first few load cycles, the value of dissipated energy decreased to stabilize finally. The first and the fifth hysteresis loops are presented in Figure 5.

### 3.4. Water Absorption

**Figure 8:**
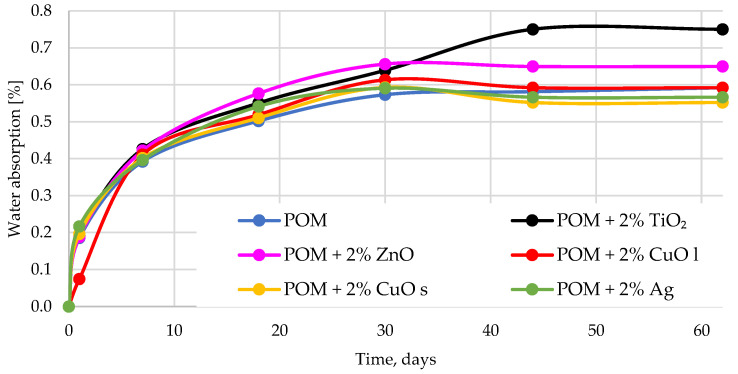
Presentation of the water absorption of composites with the addition of antibacterial particles. The index s (CuO s) stands for nanoparticles, and the index l (CuO l) stands for microparticles.

Figure 8 shows the change in water absorption in relation to incubation time. Observations showed an increase in absorbency with increasing immersion time in the water. The water absorption was at a similar level for all composites. After 30 days of incubation in water, an increase in absorbency was observed for the materials modified with zinc oxide and, more significantly, for the material modified with titanium oxide. Still, the absorbency stabilized after about 40 days of testing. The obtained results suggested that water penetrates the material, and the connection between the additive and the polymer matrix is moderate. Water penetration into composite materials occurs through one primary mechanism–diffusion. This mechanism is based on the direct diffusion of water molecules into the matrix and the particles. Other common mechanisms of moisture absorption into composite materials are capillarity and transport through microcracks. Each of them activates only after certain damage to the composites occurs. Often, this failure, which enhances moisture penetration by activating these additional mechanisms, is itself a direct consequence of exposure to moisture in the composite. The capillary mechanism involves the flow of water molecules along the modifier–matrix interface and then diffusion from this interface into the bulk of the polymer. It is not active unless the additive has been detached from the matrix, often due to water attack at the interface. Moisture transport through microcracks involves water flow and storage in microcracks or other microdamages resulting from environmental or working conditions. Activation of these mechanisms is distinguished by the increase in both the rate and maximum moisture absorption capacity in an auto-accelerating manner [53,54,55].

**Figure 9:**
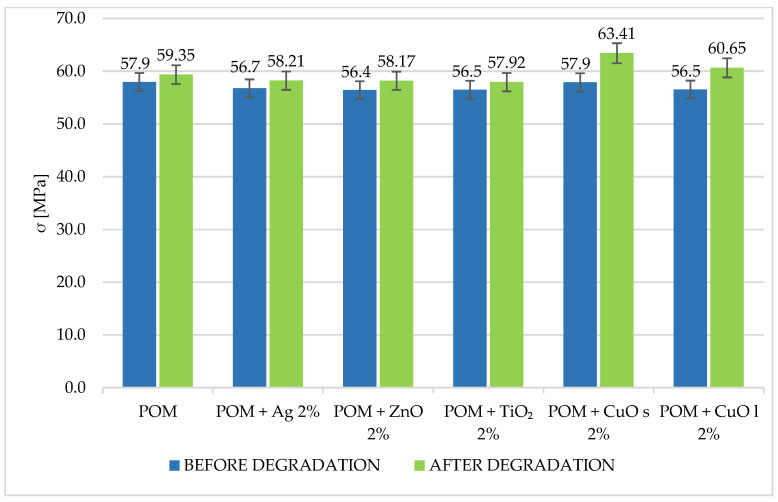
Comparison of tensile strength of tested materials before and after hydro-degradation. The index s (CuO s) stands for nanoparticles, and the index l (CuO l) stands for microparticles.

**Figure 10:**
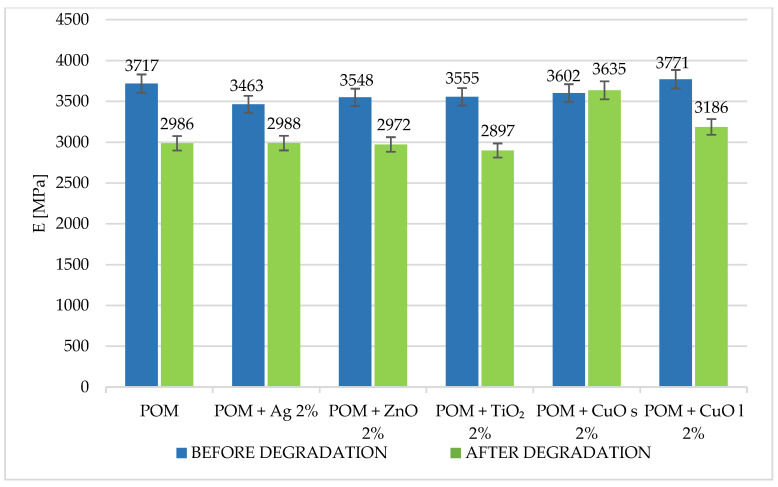
Comparison of Young’s modulus of tested materials before and after hydro-degradation. The index s (CuO s) stands for nanoparticles, and the index l (CuO l) stands for microparticles.

The strength tests after the hydrolytic degradation process were repeated to assess changes in materials. The results are presented in Figure 9 and Figure 10. For all the tested composites, the tensile strength increased by several percent, while the modulus of elasticity decreased.

Several mechanisms can characterize the effect of water on the properties of composites. In the case of polymers, the plasticization of the material associated with water penetration into the material can be seen. The reduction in the strengthening effect may be a sign of the chemical reaction phase taking place on the surface, which breaks the bonds between the matrix and the reinforcement. This effect may also be explained by the formation of a layer of water along the additives, reducing the friction between the components and, thus, the appearance of the plasticizing effect of the material. In addition, water migration into the material causes the face of the material to swell, introducing tensile stresses that can cause microcracks. Water absorption and the related swelling of the modifiers do not always result in a decrease in strength properties [55]. In the literature, examples of composites with natural fiber provided information about the positive effect of water on mechanical properties. Such an effect was reported by Dhakal et al. [56]. Studies indicate that under the influence of water absorption, the gaps between the fiber and the matrix are filled as a result of fiber swelling. This mechanism leads to an increase in the mechanical properties of the composite. The gaps that form during the production process are caused by poor reinforcement impregnation or the cured resin’s thermal shrinkage. As the fiber swells, the fiber–resin void can disappear and the fibers exert pressure on the matrix, resulting in excellent adhesion, more efficient stress transfer along the fiber–matrix interface before failure of the composite, and thus, in this case, an increase in composite strength after immersion in the water. In the case of composites containing mineral fillers, the impact of the aquatic environment demonstrates a slightly different nature due to the hydrophobic nature of mineral fibers. The tendency of the polymer to absorb moisture decreases with increasing fiber content. However, the type of fiber can change the water absorption by the presence of capillarity and affect the water content and rate of absorption.

## 4. Conclusions

In recent years, interest in materials with antibacterial properties has increased in importance. The most used additives for biocidal materials are additives based on metal ions, which damage the bacterial cell membrane and lead to its death. These types of properties are extremely desirable in construction materials that are used in the medical industry. Polyoxymethylene is one of the medical industry’s most frequently used structural polymers. In this work, polyoxymethylene was modified by silver, titanium, zinc, and copper oxide with different particle sizes. The results of strength tests did not show significant changes concerning the base material, which is a positive effect, because introducing particles to the base material often causes a decrease in tensile strength, which is associated with the formation of discontinuities. The produced composite materials were also tested for antibacterial properties. Most scientific studies focus on one or two antibacterial additives; additionally, various research methodologies are used, including methods, time, measurement conditions, or environmental conditions. The aim of this study was to check the most commonly used antibacterial additives showing potential biocidal activity under the same conditions and to identify the best ones. The results showed that titanium oxide was the best of the introduced additives, obtaining 100% biocidal effectiveness against *Escherichia coli* and about 96% effectiveness against *Staphylococcus aureus* strains. The other antibacterial additives showed a lower biocidal efficacy of about 30% against *Staphylococcus aureus*. The presented research indicates a great potential for adding titanium oxide nanoparticles to the polyoxymethylene matrix, ensuring excellent antibacterial properties while maintaining unchanged strength and functional properties.

## Figures and Tables

**Table 1:** Characteristic properties of tested polyoxymethylene Tarnoform 500 CE.

Properties	Value from Technical Data Sheet
Density (kg/m^3^)	1410
Flexural modulus, 23 °C (MPa)	2550
Ball indentation hardness, 30 s (MPa)	147
Melting temperature (°C)	166
Melt volume rate, MVR (cm^3^/10 min)	24

**Table 2:** Processing parameters.

Material	Cylinder Zone Temperatures (°C)				Mold Temperature (°C)	Pressure in the Clamping Phase (MPa)
	I	II	III	IV		
All compositions	175	185	195	200	60	100

**Table 3:** Sample description.

Sample	Description
POM	Reference sample—polyoxymethylene (POM-C; Tarnoform 500 CE)
POM + Ag 2%	Polyoxymethylene (POM-C; Tarnoform 500 CE) with 2 wt% silver (Ag) nanopowder/nanoparticles, purity: >99.995%, size: 28–48 nm, manufacturer: Nanografii (Ankara, Turkey)
POM + ZnO 2%	Polyoxymethylene (POM-C; Tarnoform 500 CE) with 2 wt% zinc oxide (ZnO) nanopowder, purity: >99.5%, size: 30–50 nm, manufacturer: Nanografii (Ankara, Turkey)
POM + TiO_2_ 2%	Polyoxymethylene (POM-C; Tarnoform 500 CE) with 2 wt% titanium (IV) oxide, 98+%, anarase powder; size: 1 µm; manufacturer: Acros Organics B.V.B.A.a part of Thermo Fisher Scientific (Waltham, MA, USA)
POM + CuO s 2%	Polyoxymethylene (POM-C; Tarnoform 500 CE) with 2 wt% copper nanooxide (CuO); nanowires; size: 40–60 nm; manufacturer: Suzhou Canfuo Nanotechnology Co., Ltd. (Suzhou, China)
POM + CuO l 2%	Polyoxymethylene (POM-C; Tarnoform 500 CE) with 2 wt% copper oxide (CuO) particles; size: 10–20 µm; manufacturer: Suzhou Canfuo Nanotechnology Co., Ltd. (Suzhou, China)

**Table 4:** Degree of reduction in *Escherichia coli* and *Staphylococcus aureus* viability.

Sample	Degree of *Escherichia coli* Viability Reduction (%)	Degree of *Staphylococcus aureus* Viability Reduction (%)
POM + Ag 2%	0	26
POM + ZnO 2%	68	29
POM + TiO_2_ 2%	100	96
POM + CuO s 2%	4	51
POM + CuO l 2%	0	26

**Table 5:** Basic mechanical properties determined in a static tensile test.

Sample	Tensile Strength σm [MPa]	Young’s Modulus E [Mpa]	Strain at Ultimate Strength [%]
POM	57.9 ± 2	3717 ± 137	8.0 ± 0.2
POM + 2% Ag	56.7 ± 1	3463 ± 155	7.9 ± 0.6
POM + 2% ZnO	56.4 ± 1	3549 ± 102	8.2 ± 0.2
POM + 2% TiO_2_	56.5 ± 0.5	3555 ± 358	8.2 ± 0.2
POM + 2% CuO s	57.6 ± 0.8	3602 ± 44	8.1 ± 0.4
POM + 2% CuO l	56.5 ± 1.1	3771 ± 74	6.9 ± 0.5

## Data Availability

Data available on request due to restrictions, e.g., privacy or ethical. The data presented in this study are available on request from the corresponding author. The data are not publicly available due to trade secrets of the company.
